# A gene associated with social immunity in the burying beetle *Nicrophorus vespilloides*

**DOI:** 10.1098/rspb.2015.2733

**Published:** 2016-01-27

**Authors:** William J. Palmer, Ana Duarte, Matthew Schrader, Jonathan P. Day, Rebecca Kilner, Francis M. Jiggins

**Affiliations:** 1 Department of Genetics, University of Cambridge, Cambridge, UK; 2 Department of Zoology, University of Cambridge, Cambridge, UK

**Keywords:** lysozyme, social immunity, burying beetle, *Nicrophorus*, parental care

## Abstract

Some group-living species exhibit social immunity, where the immune response of one individual can protect others in the group from infection. In burying beetles, this is part of parental care. Larvae feed on vertebrate carcasses which their parents smear with exudates that inhibit microbial growth. We have sequenced the transcriptome of the burying beetle *Nicrophorus vespilloides* and identified six genes that encode lysozymes—a type of antimicrobial enzyme that has previously been implicated in social immunity in burying beetles. When females start breeding and producing antimicrobial anal exudates, we found that the expression of one of these genes was increased by approximately 1000 times to become one of the most abundant transcripts in the transcriptome. Females varied considerably in the antimicrobial properties of their anal exudates, and this was strongly correlated with the expression of this lysozyme. We conclude that we have likely identified a gene encoding a key effector molecule in social immunity and that it was recruited during evolution from a function in personal immunity.

## 1. Introduction

Insects occupy some of the most microbe-rich environments in nature and have evolved diverse immunological defences to overcome the challenge that microbes pose to their fitness [1,2]. In some group-living species, individuals are selected to defend other individuals, as well as themselves, from potential pathogens. This is social immunity in the broad sense, and it is seen in transient animal societies such as animal families as well as more permanent animal societies such as the eusocial insects and group-living primates [3]. Social immunity can take a range of forms, from the collective behaviour that causes social fever in bees, to the production of antibacterial substances by parents to defend offspring or a breeding resource [2–4]. Yet, while the mechanisms underlying personal immunity in insects are increasingly well-described [5,6], relatively little is known about the mechanisms underlying social immunity (but see e.g. [7]). Nor is it clear whether social immune function might have originally been derived from personal immune function.

In burying beetles (*Nicrophorus* spp.), social immunity is a vital part of parental care. These insects breed on small vertebrate carcasses which they shave, roll into a ball and smear with anal exudates. These exudates have strong antimicrobial properties [8,9] and promote larval survival [10]. The strength of antimicrobial activity in anal exudates is proportional to the perceived microbial threat, but increasing levels of antimicrobial activity comes at a fitness cost to adults [11] and trades-off against personal immunity [12]. Antimicrobial activity in the anal exudates is thus carefully modulated. It is virtually non-existent in non-breeding individuals [9], is induced by reproduction and the presentation of a carcass [9] and reaches its maximum strength when the larvae arrive at the carcass shortly after hatching in the soil surrounding the carcass [12].

How has social immunity evolved in the burying beetle? One hypothesis is that elements of the personal immune response have been recruited to control the microbiota in the wider environment. Lysozymes, which are enzymes that can kill bacteria by hydrolysing structural polysaccharides in their cell walls, are a likely candidate because they are ubiquitous in nature and have key roles in personal immunity [5,11]. In insects that feed on microbe-rich resources (e.g. *Drosophila*, housefly), lysozymes in the gut are thought not only to have an immune function but also to digest bacteria [12,13]. Perhaps in burying beetles, lysozymes that were originally confined to the gut are now exuded and applied to the carcass to limit microbial growth during reproduction. Supporting this hypothesis is the finding that a key active antimicrobial substance in the burying beetle's anal exudates has lysozyme-like properties [10,14]

Here our aim is to test whether lysozyme genes are upregulated during the mounting of a social immune response in the burying beetle *Nicrophorus vespilloides*. We sequence the *N. vespilloides* transcriptome and identify the lysozymes within it. We then compare the transcriptional response in the gut of mated breeding females and virgin non-breeding female burying beetles to identify upregulated genes. The expression of these genes is then correlated with the antimicrobial activity of the anal exudates of different females within a population. Finally, we look at how the expression of lysozyme genes correlates changes in the lytic activity of the anal exudates throughout the breeding event of *N. vespilloides* [12].

## 2. Material and methods

### (a) Beetle rearing and dissecting

The beetles used in this experiment were bred in 2014 and descended from field-collected beetles trapped earlier that year from two sites in Cambridgeshire, UK. The field-collected beetles were interbred to create a large, genetically diverse population. This population was maintained with full parental care and no inbreeding for several generations before the start of this experiment.

We examined the transcriptional response to breeding in *N. vespilloides* by comparing the transcriptional profiles of a breeding female beetle and a non-breeding female of the same age. We focused on females alone, because our previous work suggests that they contribute more to social immunity than males [14]. Prior to each treatment, beetles were given a small meal of minced beef as part of the usual protocol for beetle husbandry in the laboratory. The ‘breeding’ treatment initially consisted of four female–male pairs of beetles. Each pair was placed in a breeding box with soil and a thawed mouse carcass (10–16 g). These boxes were then put in a dark cupboard to simulate underground conditions and the beetles were allowed to mate and begin preparing the carcass. Forty-eight hours after pairing, at peak antimicrobial activity in the anal exudates [9], we removed the female from each breeding box and placed them individually in small plastic boxes (box dimensions, length × width × depth: 12 cm × 8 cm × 2 cm) where they remained for approximately 1 h before being killed and dissected. The ‘non-breeding’ treatment consisted of four females that were treated in the same way as the ‘breeding’ treatment except that the non-breeding females were placed alone, without a male, in a breeding box that did not contain a mouse carcass. This was repeated on a second occasion with just two breeding and two non-breeding females, to generate six breeding and six non-breeding beetles. Individual beetles were euthanized with CO_2_ and immediately dissected to remove the gut. We focused on gut tissue because this is where the anal exudates are produced.

### (b) Transcriptome sequencing

The transcriptome was sequenced from a single breeding and single non-breeding female. The dissected gut was immediately homogenized in TRIzol^®^ Reagent (Life Technologies) and frozen in liquid nitrogen. RNA was extracted following the standard protocol. Illumina sequencing libraries were constructed with poly-A enrichment and sequenced in a single lane of Illumina HiSeq 2500^®^ (v. 3 chemistry, 100 bp paired-end reads) by BGI (Hong Kong). Raw reads were initially checked for quality using FastQC [15]. Having been found to be satisfactory, they were then trimmed using Trimmomatic [16], removing trailing and leading bases with a quality below q15, cutting reads where quality fell below q20 in a four base sliding window, and only retaining reads of minimum length 30.

### (c) Transcriptome assembly

The RNA-seq reads from a single breeding and a single non-breeding beetle gut were combined and the transcriptome de novo assembled (electronic supplementary material, figure S1). The assembly was performed using Trinity, a compact and fast transcript assembly program for Illumina RNA-seq data [17]. Briefly, a single Trinity assembly was built using forward and reverse reads from both libraries and default parameters. The full recommended protocol ‘Identification and Analysis of Differentially Expressed Trinity Genes and Transcripts’ was applied (http://trinityrnaseq.sourceforge.net/analysis/diff_expression_analysis.html, accessed 10 April 2015).

### (d) Differential expression analysis

Differential expression analysis was performed on the transcriptomes of a single breeding and single non-breeding female. To estimate transcript abundance, we aligned reads separately from each library onto the combined*-*read transcriptome assembly using the short read aligner bowtie [18]. Abundance estimates were then produced using RSEM [19]. These steps are combined into a single perl script bundled with Trinity, align_and_estimate_abundance.pl. In further analyses, we used estimates of transcript abundance for each gene (as opposed to each isoform). Finally, we estimated levels of differential expression using EdgeR, an R Bioconductor package for differential expression analysis. Differentially expressed transcripts were identified using the Trinity scripts run_DE_analysis.pl and analyze_diff_expr.pl with default settings. As we did not have any biological replication to estimate the amount of over-dispersion in our data, we instead fixed the over-dispersion parameter (the square-root biological coefficient of variation) to the default value of 0.1. The *p*-values will be sensitive to this parameter, so we used a very conservative significance threshold (*p* < 10^−20^, which equates to a Bonferroni corrected *p* < 8.4 × 10^−17^). Most importantly, we verified the results on which we base our conclusions by quantitative PCR (see below). In a very small number of cases, it was clear that alternative haplotypes of a gene had been split into two genes during the assembly and this gave a false signal of differential expression. To avoid this, we identified all the genes whose predicted peptides were more than 98% identical to another gene using CD-HIT [20] and excluded them from the analysis.

### (e) Peptide and domain prediction

Trinity assembles nucleotide reads into nucleotide transcripts, and as such candidate peptide sequences must be predicted post hoc (electronic supplementary material, figure S1). Peptide predictions were generated from the combined-read assembly using Transdecoder [17] and the standard protocol for peptide prediction. Any transcript that did not encode a predicted peptide was removed from our assembly.

The resulting peptide predictions were then run through the NCBI Batch Conserved Domain Search [21] to annotate domains. Putative lysozymes were then identified by the presence of the LYZ1 C-type lysozyme domain (cd00119), which is found in *Drosophila* lysozymes and expected to be required for *Drosophila-*like function of the protein.

### (f) Phylogenetics

To investigate the phylogenetic relationships of the lysozymes, we retrieved other lysozyme sequences described in previous analyses [22–24] from the NCBI protein database. Midpoint-rooted PhyML maximum-likelihood phylogeny was based on a MAAFT and GBLOCKS alignment of lysozymes and related proteins from a wide panel of taxa including both vertebrates and invertebrates. Node support values were determined from 100 bootstrap replicates, and the scale bar is substitutions per site.

### (g) Quantitative PCR

Differential expression of lysozymes was verified by quantitative PCR using six breeding and six non-breeding females. The analysis included the two individuals used in the transcriptome sequencing, but removing these samples from the statistical analysis does not alter our conclusions. We synthesized cDNA using Promega Go-script^®^ reverse transcriptase following the standard conditions using 1 µl RNA template and incubating at 25°C for 5 min, 50°C for 50 min and 70°C for 15 min. PCR primers were designed that amplify the six lysozymes (all three *Lys1* isoforms were amplified by a single primer pair) and the reference gene *actin5C* (electronic supplementary material, table S1). Quantitative PCR was performed using SYBR green using the SensiFAST SYBR Hi-ROX kit with a 10 µl reaction volume (2 µl template cDNA diluted 1 : 10 from original cDNA synthesis). Three technical replicates were performed. Differences in gene expression between the treatments were analysed using a general linear mixed model (GLMM) that included ‘experiment’ (whether the beetle was in the first or second batch) as a random effect. Significance was assessed using Wald tests.

### (h) Relationship between *Lys6* expression and lytic activity in anal exudates

In August 2015, we took 52 virgin males and females from the beetle stock population and kept them under standard stock conditions until they were sexually mature. Upon maturity, we placed females in individual plastic breeding boxes, with moist filter paper. For 3 days, we fed them daily a piece of mince (0.06–0.08 g), which was consumed within a few hours. We did this to standardize the amount of resources each female consumed prior to the breeding bout. In the afternoon of the third day, after the females had consumed the mince, we placed each female with a male in a breeding box half-filled with moist compost. Each pair was provided with a mouse carcass and allowed to prepare it.

Approximately 42 h after pairing, we collected anal exudates from females. Beetles produce exudates readily when tapped gently on the abdomen, but in one case exudate was not produced in enough volume and this female was excluded from the dataset. Exudates were diluted to a concentration of 1 : 5 in 0.2 M pH 6.4 potassium phosphate buffer and kept at −20°C until further analysis. We then anaesthetized females with CO_2_ and dissected their guts, which were immediately homogenized in TRIzol^®^ reagent (Life Technologies) and frozen in liquid nitrogen for later RNA extraction quantitative PCR was used to quantify expression of all lysozyme genes.

We performed a lytic zone assay to measure antimicrobial activity in anal exudates following Cotter *et al*. [8]. In brief, agar was mixed with a solution of frozen *Micrococcus lysodeikticus* cells and plated in Petri dishes. We punched holes of approximately 1 mm diameter into the solidified agar mix and applied 1 µl of thawed exudate in each hole, with two technical replicates per sample. We measured the diameter of the lytic zone appearing after 24 h of incubation at 33°C, using the software ImageJ. Egg white lysozyme at known concentrations was also applied in holes to create standard curves from which we derived the slope and intercept of the regression explaining the relationship between lytic activity (in mg ml^−1^ lysozyme equivalents) and diameter of the lytic zone.

After inspection of the data, we identified three outliers which were subsequently removed from the analysis. We excluded another female because her brood failed and no antimicrobial activity was present in her exudates. We estimated the correlation between the log_2_ transformed measurements of lytic activity and relative gene expression using a GLMM. The response was the technical replicates of both the qPCR and lytic zone assay. The type of measurement was a fixed effect (qPCR or lytic zone). We estimated separate residuals and the covariance and variance of the qPCR or lytic zone measurements. The model parameters were estimated using the R package MCMCglmm [25].

### (i) *Lys6* expression throughout the breeding bout

A further 50 beetle pairs were established in September 2015 following the standard breeding protocol to examine gene expression in females at different stages of the breeding bout. We removed females at days 1, 4 and 8 after pairing and dissected their gut for later RNA extraction. We used quantitative PCR to measure expression of all lysozyme genes. We only used females that showed no sign of brood failure (day 1: *N* = 14, day 4: *N* = 15, day 8: *N* = 16).

Analysis of relative gene expression for each lysozyme gene was done with a GLMM, with female's family of origin as a random effect and days after pairing as a fixed effect. Model parameters were estimated using the R package lme4. Tukey post hoc comparisons were performed using the R package lsmeans.

## 3. Results

### (a) The burying beetle transcriptome

**Figure 1.**
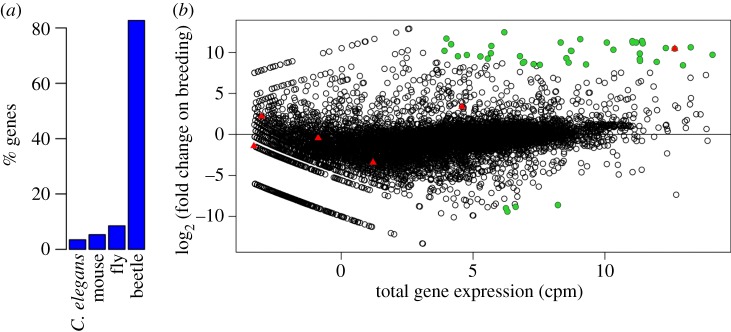
The transcriptome of *N. vespilloides.* (*a*) The percentage of peptides whose most similar sequence was in the genome of the mouse *M. musculus*, the nematode *C. elegans*, the fly *D. melanogaster* or the beetle *T. castaneum.* A single isoform of each gene in the differential expression analysis is included. (*b*) Total gene expression (counts per million) and log_2_ (fold change) in gene expression in the guts of breeding versus non-breeding females. Lysozymes are shown in red triangles. The most significantly differentially expressed genes (*p* < 10^−20^, Bonferroni corrected *p* < 8.4 × 10^−17^) are in green circles. (Online version in colour.)

To allow us to investigate the transcriptional response in the guts of burying beetle when they breed, we first sequenced the transcriptome from the guts of a single breeding and a single non-breeding beetle, combined the sequence reads and then assembled them de novo*.* This process resulted in 11 290 genes that encoded 26 378 different transcripts. This suggests that we sequenced the majority of genes in the genome, as the exceptionally well-annotated *Drosophila* genome contains 13 920 protein coding genes encoding 30 443 transcripts (Flybase release 6). As the guts we used for the RNA extraction might contain poly-adenylated RNA from the mouse the beetles were feeding on, or nematode parasites, we used Blast to search for the most similar sequence in the *Mus musculus*, *Caenorhabditis elegans*, *Drosophila melanogaster* and *Tribolium castaneum* genomes. The top hit of 91% of the genes was another insect (*Drosophila* or the beetle *Tribolium*), suggesting the levels of contamination were low (figure 1*a*).

### (b) Many genes are strongly upregulated in the guts of breeding beetles

By mapping reads from the breeding and non-breeding beetles to the transcriptome, we found that there was a strong transcriptional response in the breeding beetles (figure 1*b*). Among the most significantly differentially expressed genes (*p* < 10^−20^, Bonferroni corrected *p* < 8.4 × 10^−17^), 90% were upregulated in the breeding beetles (figure 1*b*; *N* = 42, 95% binomial CI: 77–97%). The magnitude of these changes in transcription was often large—on average the expression of these 42 most significantly differentially expressed genes changed by nearly 1000 times (mean log_2_ (fold change) = 9.96). Furthermore, some of the most strongly differentially expressed genes also had the highest total levels of expression in our transcriptome (figure 1*b*).

Several of the 42 most significantly differentially regulated genes may play a role in immunity. Based on conserved domains and/or the top *Drosophila* blast hit, 10 were serine proteases and one was a serine protease inhibitor (serpin; electronic supplementary material, table S1). These genes play a key role in regulating insect immune responses as well as other functions [26]. Other likely immune genes included a peptidoglycan recognition protein, a Toll receptor, a C-type lectin and a homologue of CG10960, which is thought to regulate the JAK-STAT pathway in *Drosophila* [27].

### (c) A lysozyme is highly expressed in breeding females

**Figure 2.**
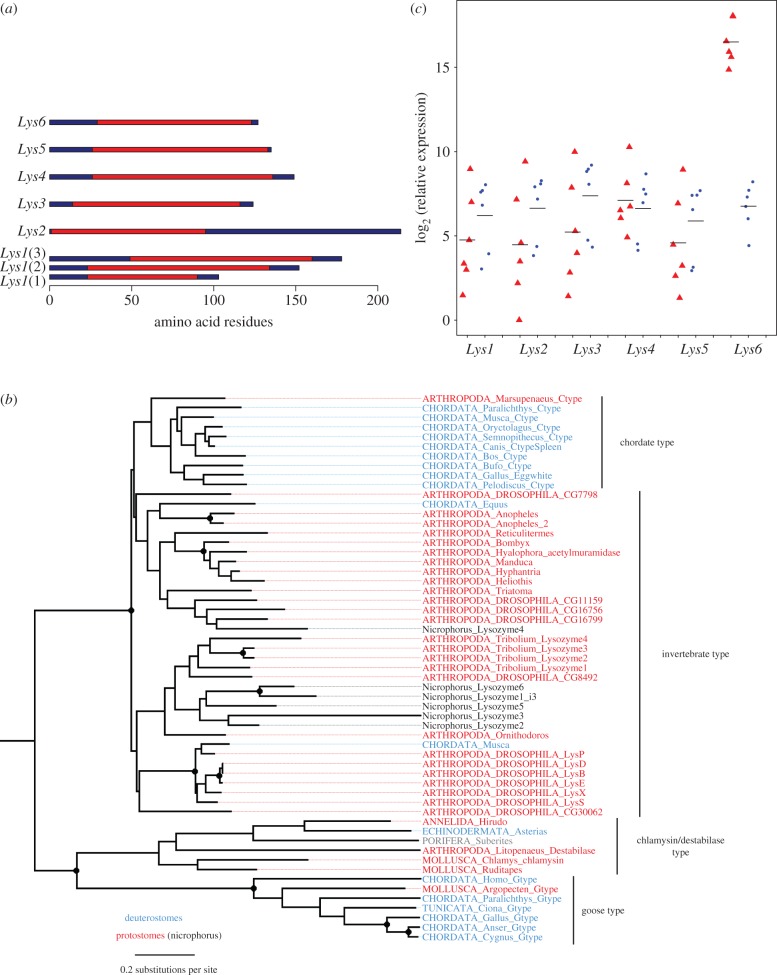
Lysozymes and their expression. (*a*) The six predicted lysozymes in the transcriptome of *N. vespilloides.* The LYZ1 C-type lysozyme domain (cd00119) is shown in red. There are three alternative isoforms of *Lys1.* (*b*) Phylogenetic relationship of lysozymes from *N. vespilloides* and other species. Bootstrap support more than 90% is indicated with a filled circle (full bootstrap results are available on Dryad). (*c*) The expression of the lysozyme genes in the guts of six breeding (red triangles) and six non-breeding (blue circles) females. Expression was measured by quantitative PCR relative to *Actin5C* (scale shifted so begin at zero)*.* Each point is the mean of three technical replicates and the horizontal bars are means. (Online version in colour.)

We identified lysozymes by searching for the conserved LYZ1 domain, which contains the active site of C-type lysozymes. Using this approach, we identified six lysozymes (figure 2*a*). These ranged in size from 103–214 amino acids, which is within the typical size range of insect lysozymes. We aligned these protein sequences with lysozymes from other organisms and reconstructed their phylogeny (figure 2*b*). All six were Invertebrate-type lysozymes, which are the commonest class of lysozymes in arthropods (figure 2*b*). While bootstrap support for the relationships is low, five of the lysozymes appear to have arisen by gene duplication during the evolution of beetles, while *Lys4* falls in a different clade that likely diverged early in insect evolution (figure 2*b*).

To identify the gene that may be responsible for the antimicrobial activity of the anal exudate of breeding females, we compared the expression of the six lysozyme genes in breeding and non-breeding females in the whole transcriptome data. Five of the genes had similar expression levels in breeding and non-breeding beetles, while *Lys6* was massively upregulated—the expression level in the breeding female was 1409 times greater than in the non-breeding female (figure 1*b*; log_2_ (fold change) = 10.46, *p* < 10^−26^, Bonferroni corrected *p* < 10^−25^). In the breeding beetle, *Lys6* was the 14th most abundant transcript in the entire transcriptome, whereas in the non-breeding beetle it was only the 5967th most abundant.

We confirmed this result using quantitative PCR to measure the expression of the lysozymes across the six breeding and six non-breeding females (figure 2*c*). In the non-breeding females, the different lysozymes all had similar levels of expression. As was the case in the transcriptome analysis, *Lys6* was strongly upregulated in breeding females, with an average expression level that was 860 times than non-breeding beetles (figure 2*c*; Wald test: *χ*^2^ = 160, d.f. = 1, *p* < 10^−16^, Bonferroni corrected *p* < 10^−15^). The expression of the five remaining lysozymes was unaltered in the breeding females (figure 2*c*).

### (d) Lysozyme expression is correlated with antimicrobial activity

**Figure 3.**
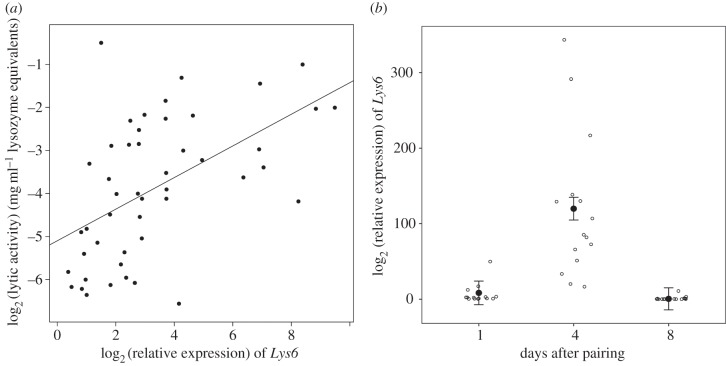
Relationship between *Lys6* expression and the phenotype. (*a*) The correlation of *Lys6* expression and lytic activity in beetle anal exudates (*N* = 47). Expression was measured by quantitative PCR relative to *Actin5C*. Lytic activity of exudates was measured in a lytic zone assay relative to known concentrations of hen egg white lysozyme. The values plotted correspond in both axes to the mean of two technical replicates. (*b*) Change of *Lys6* expression throughout the breeding bout. Expression of *Lys6* was significantly higher on day 4 than on day 1 (post hoc Tukey comparison: estimated difference = 111.47, *p* < 0.0001) and day 8 (post hoc Tukey comparison: estimated difference = 119.38, *p* < 0.0001). Bonferroni correcting these *p*-values for the six genes investigated yields *p* < 0.001 in all cases. Black circles show least-squares means of a linear mixed model with standard error bars. White circles show data points corresponding to each day, jittered to avoid overlap.

To investigate whether lysozyme is an effector molecule in the social immune defences of burying beetles, we tested whether lysozyme gene expression is correlated with the antimicrobial activity of the anal exudates across individuals. There was considerable variation in lytic activity, equivalent to over a 100-fold difference in lysozyme activity between samples (figure 3*a*). Across breeding females, we found a positive correlation between lytic activity and *Lys6* mRNA levels (figure 3*a*). The correlation (the proportion of variance in common between the traits) was 0.55 (95% credible interval: 0.33–0.75; estimated using a GLMM). After correcting for multiple tests, there was no correlation between the expression of other lysozyme genes and lytic activity (electronic supplementary material, figure S1).

The production of antimicrobial exudates changed considerably through the breeding event. Antimicrobial activity increases during the first 4 days, reaching a peak at the time of larval hatching between days 3 and 4 after pairing, and subsequently declines [10,12]. To investigate whether expression of any of the lysozyme genes followed the same pattern, we quantified gene expression, using qPCR, in females at different stages of the breeding bout. Of the six lysozyme genes, we found that only *Lys6* expression changed significantly throughout the breeding event. On day 4 after pairing, *Lys6* expression was significantly higher than on days 1 and 8 (figure 3*b*; Tukey post hoc comparison: day 4–day 1: 111.47, s.e. = 20.02, *t*_33_ = 5.56, *p* < 0.0001; day 4–day 8: 119.38, s.e. = 19.44, *t*_34_ = 6.14, *p* < 0.0001; day 1–day 8: 7.90, s.e. = 19.86, *t*_35_ = 0.39, *p* = 0.91). None of the other lysozyme genes changed expression levels throughout the breeding bout (electronic supplementary material, figure S2).

## 4. Discussion

Our analyses indicate that breeding induces a very strong transcriptional response in female burying beetles, causing substantial upregulation of just one lysozyme gene (*Lys6*) in their gut tissues relative to non-breeding females. We found that breeding females varied considerably in the expression of this gene, and we show that this is correlated with variation in the antimicrobial properties of their anal exudates. Furthermore, *Lys6* expression peaked around larval hatching when offspring are most dependent on parental care and the antimicrobial activity of the exudates is greatest [9,10]. Together with the previous observations that the exudates have lysozyme activity, these results together strongly suggest that upregulation of *Lys6* causes at least some of the change in the exudates' antimicrobial properties during breeding.

The finding that a lysozyme has a role in social immunity is not surprising because these enzymes are secreted onto external surfaces that are vulnerable to infection, such as the gut, eyes, mucous membranes and respiratory tract, providing a broad-spectrum defence against microbes in the environment [12]. It may therefore be straightforward to recruit lysozymes to social immune functions. By choosing to focus on lysozyme genes here, we were able to gain two novel insights which might otherwise not have been possible. First, we were able to show that continuous variation in *Lys6* gene expression is associated with continuous variation in the phenotype, measured as lytic activity in anal exudates. This is a more detailed and quantitative description of gene function than has been previously been possible in burying beetles [28], or indeed many other non-model organisms. Second, since insects typically possess multiple lysozyme genes with diverse functions, analyses of lysozyme sequences allowed us to infer the evolutionary relationships among them and therefore to deduce the evolutionary origin of any gene(s) associated primarily with social immune function. We found that *Lys6* is closely related to other lysozymes in the genome, providing evidence to support the hypothesis that burying beetles have recruited a component of their personal immune system to play a major role in social immunity.

Nevertheless, it is likely that other genes also contribute to social immunity in the burying beetle, and we found that several other genes with potential immune functions were also upregulated during breeding (extending similar findings previously obtained by Parker *et al*. [28]). Previous work indicates that the chemical composition of *N. vespilloides*' anal exudates is complex [29]. For example, Degenkolb *et al*. [29] identified several substances (though not lysozyme) with potential antimicrobial and antifungal properties in exudates of non-breeding beetles. However, apart from the identification of lysozyme in exudates of breeding beetles, any changes in the chemical composition of the exudates that may be induced by breeding have not previously been as thoroughly characterized, nor is it clear whether gut symbionts are involved in the production of some of the other components previously found in the exudates.

It might be argued that bacteria form a key part of the diet of breeding burying beetles or their larvae, but not of non-breeding burying beetles. Thus, a possible alternative interpretation of our data is that the increased expression of *Lys6* primarily serves a digestive function, rather than an immune function, as has been suggested for the lysozymes expressed in housefly or *Drosophila* guts. However, we think this alternative interpretation is unlikely as behavioural evidence suggests that beetles prefer to feed on meat rather than on the microbes living on the meat [7]. Furthermore, beetles in both treatments were fed meat before the experiment, whether they bred or not, which suggests that upregulation of *Lys6* in the breeding beetles was not induced simply to aid digestion. Thus, although at this stage we cannot rule out the possibility that the large increase in *Lys6* expression plays some minor role in digestion, this is unlikely to be its sole or even primary function.

A further alternative interpretation of our data is that the changes we detected in lysozyme gene expression during reproduction might be attributable to mating alone, rather than any social immune function. There is evidence from several insect species that the act of mating is sufficient to induce changes in immunity. For example, in *D. melanogaster*, mating causes increased expression of some immunity genes, while downregulating others [30,31]. In *Gryllus texensis* crickets, mating increases resistance to bacterial infections [32]. Yet in several other invertebrate species such as mealworms [33], damselflies [34], ground crickets [35] and moths [36], mating suppresses immune responses, at least partly. In the female burying beetle, mating without a carcass increases phenoloxidase (PO) activity in the haemolymph—a commonly measured part of the invertebrate personal immune response—whereas mating on a carcass suppresses PO activity [37]. As for lytic activity in anal exudates, mating in the absence of a carcass leads to a slight increase in lytic activity, but to a much smaller extent than when a carcass is also presented [9]. Thus, while it is possible that mating alone contributed to some of the upregulation of *Lys6* expression, presentation of the carcass, and the associated need to defend it from microbial attack, probably accounted for the majority of the increase in this gene's transcription that we found during reproduction.

Killing microbes in the environment is important for many insects, and a diverse range of different mechanisms has evolved. Just as with burying beetles, the antimicrobial agents are provided by the parent in European beewolfs (*Philanthus triangulum*). These hunting wasps place a paralysed bee in a brood cell and transfer symbiotic bacteria from glands on their antennae to the brood cell at the same time as laying eggs [38]. These symbionts are thought to produce antibiotic compounds that protect against fungal infection [38]. The mother also stops the paralysed bee from going mouldy by wrapping it in a secretion that keeps it dry by preventing water condensing [39]. Similarly, larvae of the emerald cockroach wasp (*Ampulex compressa*) develop on cockroaches (*Periplaneta americana*) and produce antimicrobial oral secretions that kill bacteria growing in their host [40]. Unlike burying beetles, these antimicrobials do not appear to have been recruited from the conventional insect immune system as the active components—(R)-(-)-mellein and micromolide—are not known to be have antimicrobial functions in other insects [40].

In summary, we have found a gene (*Lys6*) associated with social immunity in the burying beetle, together with evidence that it was recruited from personal immune function in the evolutionary past. The challenge for future work is to determine how this gene's function is integrated with other components of the social immune system to influence the microbial community on the burying beetle's breeding resource.

## Supplementary Material

Supplementary Table 1

## Supplementary Material

Supplementary Figures 1–2

## Data accessibility

The raw sequence data have been submitted to the NCBI sequence read archive under the accession nos. SRR2071758 (breeding) and SRR2071757 (non-breeding). This Transcriptome Shotgun Assembly project has been deposited at DDBJ/EMBL/GenBank under the accession no. GDUU00000000. The version described in this paper is the first version, GDUU01000000. The predicted lysozyme sequences have been submitted to GenBank under the accession nos. KU060803-KU060810. Predicted peptides, full domain annotations and the raw data used to generate the figures have been submitted to Dryad http://dx.doi.org/10.5061/dryad.6n4fm.
