# Supplementary material for: A gene associated with social immunity in the burying beetle Nicrophorus vespilloides
Source: Proc Biol Sci. 2016 Jan 27;283(1823):20152733. doi: 10.1098/rspb.2015.2733 (PMC4795035; doi:10.1098/rspb.2015.2733)
Supplement: Supplementary Figures 1–2 [file rspb20152733supp2.docx]

**
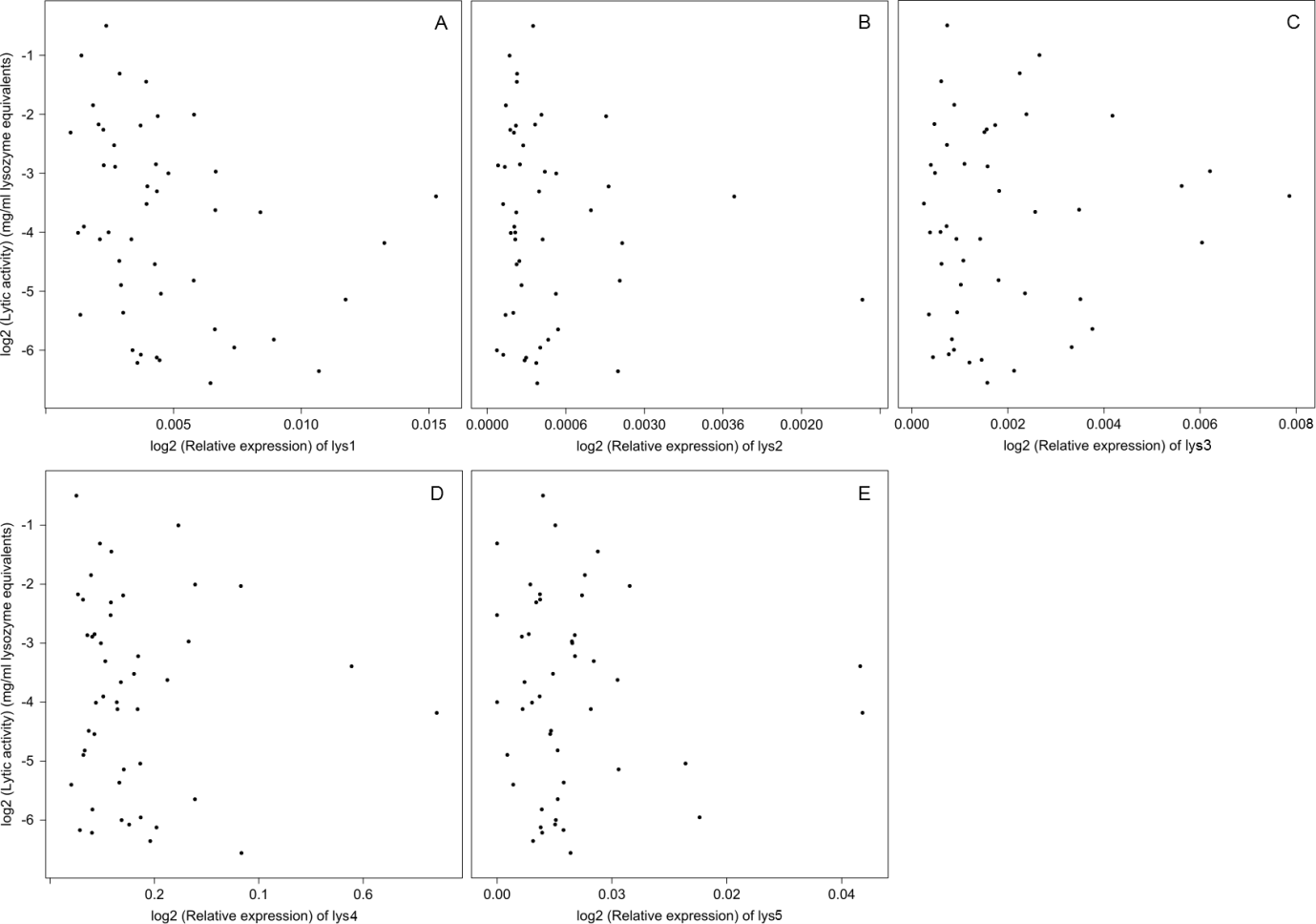
**

**Supplementary Figure 1.** Relative expression of lysozyme genes 1-5 does not correlate significantly with lytic activity in beetle anal exudates, after Bonferroni correction (Pearson product-moment correlations, *Lys1*: *t*_45_= -2.15, *p* = 0.22; *Lys2*: *t*_45_ = -0.89, p= 1.00; *Lys3*: *t*_45_ = 0.44, *p* = 1.00; Lys4: *t*_45_ = -0.29, *p* = 1.00; Lys5: *t*_45_ = -0.37, *p* = 1.00). Expression was measured by quantitative PCR relative to *Actin5C*. Lytic activity of exudates was measured in a lytic zone assay relative to known concentrations of hen egg white lysozyme. The values plotted correspond in both axes to the mean of two technical replicates.

**
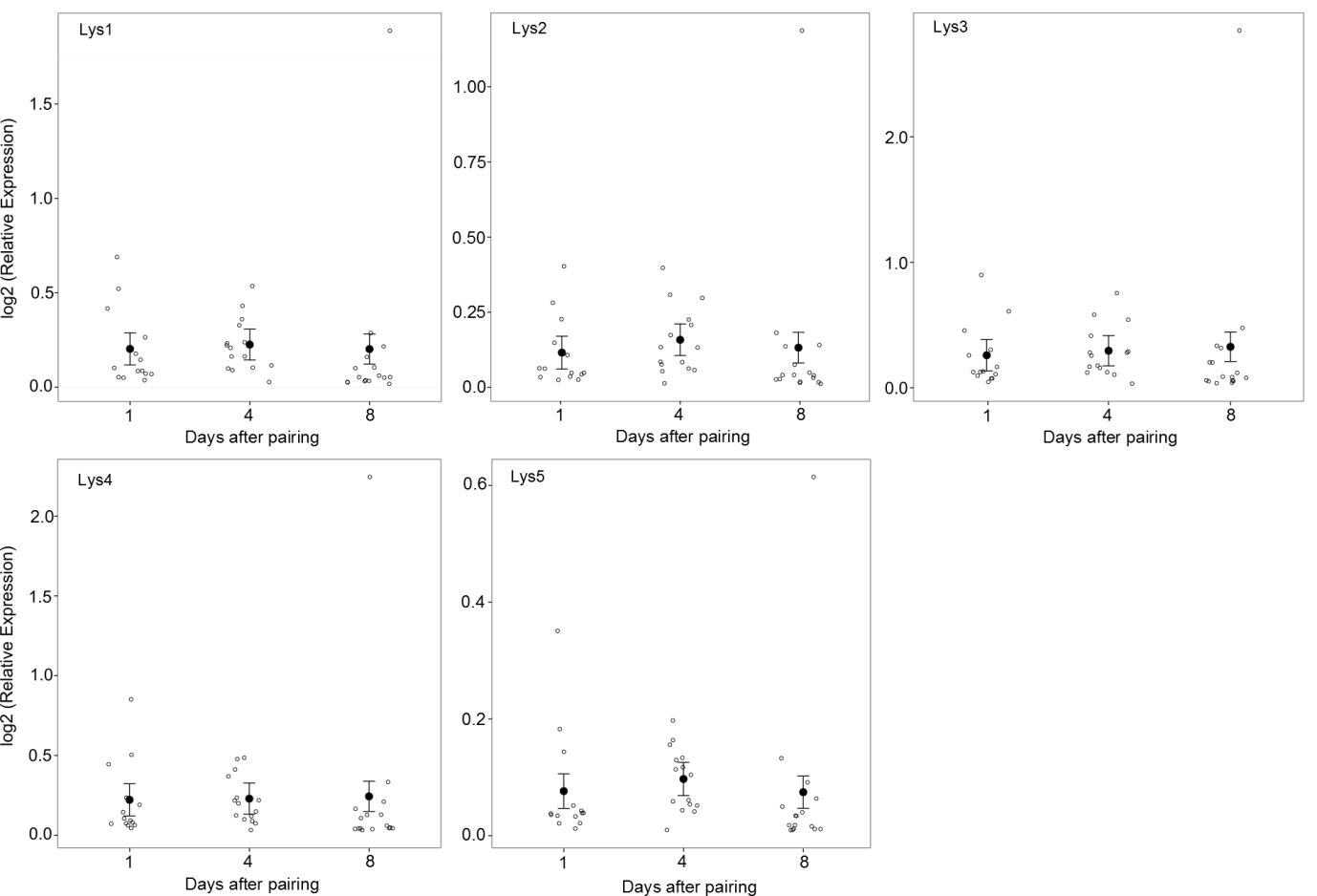
**

**Supplementary Figure 2.** Relative expression of lysozyme genes 1-5 (indicated in top left corner of each panel) throughout the breeding bout in female beetles. Expression was measured by quantitative PCR relative to *Actin5C*. Expression did not change significantly throughout the breeding bout for any of the 5 shown lysozyme genes (GLMM’s with relative expression as response variable and ‘Days after pairing’ as fixed effect: *p*-values > 0.5 in all five models; female family of origin included as random effect). Black circles show least-squares means of a linear mixed model with standard error bars. White circles show data points corresponding to each day, jittered to avoid overlap.
